# Sequencing‐based microsatellite instability testing using as few as six markers for high‐throughput clinical diagnostics

**DOI:** 10.1002/humu.23906

**Published:** 2019-09-15

**Authors:** Richard Gallon, Harsh Sheth, Christine Hayes, Lisa Redford, Ghanim Alhilal, Ottilia O'Brien, Helena Spiewak, Amanda Waltham, Ciaron McAnulty, Osagie G. Izuogu, Mark J. Arends, Anca Oniscu, Angel M. Alonso, Sira M. Laguna, Gillian M. Borthwick, Mauro Santibanez‐Koref, Michael S. Jackson, John Burn

**Affiliations:** ^1^ Institute of Genetic Medicine Newcastle University Newcastle upon Tyne United Kingdom; ^2^ FRIGE's Institute of Human Genetics FRIGE House Ahmedabad India; ^3^ Northern Genetics Service Newcastle Hospitals NHS Foundation Trust Newcastle upon Tyne United Kingdom; ^4^ Division of Pathology, Institute of Genetics & Molecular Medicine University of Edinburgh Edinburgh United Kingdom; ^5^ Department of Molecular Pathology, Laboratory Medicine Royal Infirmary of Edinburgh Edinburgh United Kingdom; ^6^ Oncogenetics and Hereditary Cancer Group, Navarrabiomed, Complejo Hospitalario de Navarra (CHN), Instituto de Investigación Sanitaria de Navarra (IdiSNA) Universidad Pública de Navarra (UPNA) Pamplona Spain

**Keywords:** colorectal cancer, high‐throughput diagnostics, microsatellite instability, mismatch repair deficiency, single‐molecule molecular inversion probes

## Abstract

Microsatellite instability (MSI) testing of colorectal cancers (CRCs) is used to screen for Lynch syndrome (LS), a hereditary cancer‐predisposition, and can be used to predict response to immunotherapy. Here, we present a single‐molecule molecular inversion probe and sequencing‐based MSI assay and demonstrate its clinical validity according to existing guidelines. We amplified 24 microsatellites in multiplex and trained a classifier using 98 CRCs, which accommodates marker specific sensitivities to MSI. Sample classification achieved 100% concordance with the MSI Analysis System v1.2 (Promega) in three independent cohorts, totaling 220 CRCs. Backward–forward stepwise selection was used to identify a 6‐marker subset of equal accuracy to the 24‐marker panel. Assessment of assay detection limits showed that the 24‐marker panel is marginally more robust to sample variables than the 6‐marker subset, detecting as little as 3% high levels of MSI DNA in sample mixtures, and requiring a minimum of 10 template molecules to be sequenced per marker for >95% accuracy. *BRAF* c.1799 mutation analysis was also included to streamline LS testing, with all c.1799T>A variants being correctly identified. The assay, therefore, provides a cheap, robust, automatable, and scalable MSI test with internal quality controls, suitable for clinical cancer diagnostics.

## 1 INTRODUCTION

Increased microsatellite instability (MSI) is a hallmark of mismatch repair (MMR) deficiency, which affects approximately one in six colorectal cancers (CRCs; Boland et al., 1998). Lynch syndrome (LS) is an inherited predisposition to cancer caused by germline pathogenic variants affecting one allele of an MMR gene and accounts for approximately one in five MMR deficient CRCs (Hampel et al., 2008). MMR deficiency is also associated with tumor response to immune checkpoint blockade therapy, irrespective of tissue of origin (Le et al., 2017). Therefore, the assessment of MSI, or MMR, status can inform patient management and is recommended in all CRCs by national and international guidelines to screen for LS (Balmana, Balaguer, Cervantes, & Arnold, 2013; Newland et al., 2017; Stoffel et al., 2015). Once identified, patients with LS benefit from surveillance colonoscopy, prophylactic surgery, and chemoprevention (Burn et al., 2011; Vasen et al., 2013).

MMR status of tumors is commonly assessed by immunohistochemistry (IHC) of MMR proteins, or polymerase chain reaction (PCR) fragment length analysis (FLA) of microsatellites to detect increased MSI. MMR deficiency is inferred from the absence of at least one MMR protein, or high levels of MSI (MSI‐H). MSI‐H is defined by mutation of ≥30–40% of microsatellites analyzed (Boland et al., 1998). These methods are highly sensitive and specific, with reported sensitivities and specificities of 93% and 95% for IHC of all four MMR proteins (Shia, 2008), and 97% and 100% for FLA of mononucleotide repeats (MNRs; Bacher et al., 2004). IHC and FLA also perform well with respect to other demands of diagnostic tests. FLA is considered highly reproducible, with 98% concordance of results observed between independent laboratories (Zhang, 2008), although IHC shows some heterogeneity due to discordant interpretation of variable staining, and use of different antibodies (Shia, 2008). FLA has been shown to be reliable when sample tumor cell content is ≥10% (Berg et al., 2000), and IHC can detect focal MMR deficiency (Chapusot et al., 2002). Both are also considered to be relatively cheap and cost‐effective for LS screening (Snowsill et al., 2014). However, the uptake of MMR deficiency testing has been poor; only 28% of 152,993 CRC cases were analyzed during 2010–2012 in the USA (Shaikh, Handorf, Meyer, Hall, & Esnaola, 2018), with a similar proportion being analyzed in the UK This is despite guidelines recommending testing and estimates that only 1.2% of LS gene carriers were known to clinical services in the US in 2011 (Hampel & de la Chapelle, 2011). We estimate that only 5% of carriers are currently known in the UK.

Automated sequence analysis is better suited to high‐throughput diagnostics than FLA, or IHC, leading to the development of next‐generation sequencing (NGS)‐based MSI assays that analyze microsatellites captured by gene panel sequencing. These determine the mutation status of each microsatellite from the frequency of length variants detected, and then use the proportion of microsatellites that are mutated to classify a sample. Several such classifiers have reported sensitivities and specificities >95% (Kautto et al., 2016; Zhu et al., 2018), and have identified samples misclassified by conventional MMR deficiency tests, highlighting that there is no gold standard reference method (Hause, Pritchard, Shendure, & Salipante, 2016). Gene panel sequencing also allows additional clinically actionable markers to be simultaneously assessed. For example, separate testing for the *BRAF* c.1799T>A variant (p.V600E), following FLA or IHC, is recommended to increase the specificity of LS screening (Newland et al., 2017), but both MSI and *BRAF* can be analyzed by a single tumor sequencing assay (Hampel et al., 2018). However, the high cost of gene panel sequencing (Marino et al., 2018) may be a barrier to its widespread deployment for MSI testing, or for the detection of LS by MMR gene sequencing. Targeted NGS‐based MSI assays that use multiplex amplification of specific panels of microsatellites have been developed that, similar to gene panel‐based methods, classify samples by the proportion of microsatellites that are mutated (Gan et al., 2015; Hempelmann et al., 2018; Hempelmann, Scroggins, Pritchard, & Salipante, 2015; Waalkes et al., 2018). However, even when using the same method, different marker proportions can be used as a classification threshold with different marker sets (Hempelmann et al., 2015; Hempelmann et al., 2018; Kautto et al., 2016; Waalkes et al., 2018; Zhu et al., 2018), and thresholds can be uncertain when relatively few microsatellites (<20) are analyzed (Hempelmann et al., 2015).

We have previously used amplicon sequencing of short (7–12 base pairs [bp]), monomorphic MNRs to classify the MSI status of CRCs, without needing matched normal tissue (Redford et al., 2018). Short MNRs were selected as longer (>15 bp) microsatellites are associated with increased PCR and sequencing error (Fazekas, Steeves, & Newmaster, 2010), and it has been reported that 9–15 bp microsatellites give the greatest differences in mutation frequencies between MSI‐H and microsatellite stable (MSS) samples using NGS (Maruvka et al., 2017). Our method for MSI detection accounts for the individual sensitivity and specificity of each marker, and achieved >97% accuracy in 209 CRCs with only 17 markers, using FLA as the reference method (Redford et al., 2018). However, the protocol required singleplex PCR amplification, followed by the second round of PCR to prepare the amplicons for sequencing. Here, we modify this method to develop an MSI assay suitable for clinical cancer diagnostics. We use single‐molecule molecular inversion probes (smMIPs; Hiatt, Pritchard, Salipante, O'Roak, & Shendure, 2013) to amplify in multiplex and sequence 24 short MNRs, and show that the assay achieves 100% accuracy with as few as six markers. We also include *BRAF* c.1799 sequencing for streamlined LS screening (Newland et al., 2017). To establish the assay is suitable for clinical practice, we follow joint guidelines from the Association for Molecular Pathology and the College of American Pathologists (Jennings et al., 2017). This includes validation of diagnostic accuracy using independent sample cohorts, assessment of reproducibility and detection limits, the definition of quality control criteria, and deployment in an independent diagnostic laboratory.

## 2 MATERIALS AND METHODS

Unless stated otherwise, the manufacturer's protocols were followed for each kit or reagent.

### 2.1 Patient samples

Nineteen and 73 CRC DNAs were provided by the Department of Molecular Pathology, University of Edinburgh, UK, and the Oncogenetics and Hereditary Cancer Group, Complejo Hospitalario de Navarra, Spain, respectively. These 92 samples were residual stocks from Redford et al. (2018). An additional 128 CRC DNAs or CRC formalin‐fixed paraffin‐embedded (FFPE) tissue samples were provided by the Northern Genetics Service, Newcastle Hospitals NHS Foundation Trust, UK. Nineteen DNAs extracted from peripheral blood leukocytes (PBLs), from patients consenting to sample‐use in assay development, were gifted by K. Wimmer (Medical University of Innsbruck, Austria) and used as MSS controls.

All CRC samples (Table S1) were independently tested for MMR deficiency by the contributing laboratory using the MSI Analysis System v1.2 (Promega); samples with one mutant marker (MSI low) were considered equivalent to MSS samples (Halford et al., 2002). Forty‐six of the MSI‐H samples were independently tested for *BRAF* c.1799T>A by high‐resolution melt (HRM) curve analysis (Nikiforov et al., 2009) on a LightCycler 480 (Roche), as part of the LS screening pipeline at the Northern Genetics Service.

All samples were anonymized by the contributing laboratory, and analyzed following approval by the NHS Health Research Authority Research Ethics Committee (13/LO/1514).

### 2.2 Cell lines and culture

H9 embryonic stem cell line (WiCell, agreement number 06‐W097) DNA was a gift from L. Lako (Newcastle University, UK), and used as an MSS control. HCT116 (CCL‐247; ATCC) and K562 (CCL‐243; ATCC) cells were gifted by J. Irving (Newcastle University, UK). HCT116 and K562 cells were grown in the Roswell Park Memorial Institute growth medium containing 2 mM l‐glutamine (Gibco), 10% fetal bovine serum (Gibco), 60 µg/ml penicillin, and 100 µg/ml streptomycin (Gibco) at 37°C and 5% CO_2_. HCT116 cells were passaged or harvested at 80–90% confluence by decanting expired growth medium, washing in 5 ml phosphate‐buffered saline (Gibco), and detaching the cells using 0.05% trypsin–ethylenediaminetetraacetic acid (Gibco). K562 cells were passaged or harvested at a density of 1 × 10^6^cells/ml. DNA extracted from HCT116 CRC cell line (MLH1 deficient) was used as an MSI‐H control. DNA extracted from K562 chronic myeloid leukemia cell line was used as an MSS control.

### 2.3 Sample mixtures and dilutions

Mixtures of MSI‐H and MSS samples were created using HCT116 and PBL DNAs (Table 1). Nine samples, comprising three fresh tissues (HCT116, H9, and K562 cell lines), and six FFPE tissues (three MSI‐H CRCs: N021, N068, and N073; and three MSS CRCs: N033, N036, and N056), were twofold serially diluted in 10 mM Tris‐HCl pH 8.5.

**Table 1:** Generation of sample mixtures with varying MSI‐H content

MSI‐H cell line DNA content (%)	Mixture of DNAs (25 ng/µl)
50.00	10 µl of HCT116 DNA + 10 µl of PBL DNA
25.00	10 µl of 50.00% mixture + 10 µl of PBL DNA
12.50	10 µl of 25.00% mixture + 10 µl of PBL DNA
6.25	10 µl of 12.50% mixture + 10 µl of PBL DNA
3.13	10 µl of 6.25% mixture + 10 µl of PBL DNA
1.56	10 µl of 3.13% mixture + 10 µl of PBL DNA
0.78	10 µl of 1.56% mixture + 10 µl of PBL DNA

Abbreviations: MSI, microsatellite instability; MSI‐H, high levels of MSI; PBL, peripheral blood leukocyte.

### 2.4 DNA extraction and quantification

DNA was extracted from FFPE CRC tissue using the GeneRead DNA FFPE Kit (Qiagen). DNA was extracted from cell lines using the Wizard Genomic DNA Purification Kit (Promega). DNAs were quantified using QuBit 2.0 Fluorometer (Invitrogen) and QuBit dsDNA BR/HS Kits (Invitrogen).

### 2.5 Markers and smMIP design

The marker panel includes 24 MNRs, previously published by Redford et al. (2018), for MSI classification, as well as *BRAF* c.1799 to screen for sporadic MSI‐H CRCs (Newland et al., 2017). MIPgen (Boyle, O'Roak, Martin, Kumar, & Shendure, 2014) was used to generate smMIP sequences for each marker. MIPgen parameters were: Tag size 6, 0, minimum capture size 120, and maximum capture size 150. smMIP designs were selected by the following criteria: No common single nucleotide polymorphisms (SNPs) in the smMIP extension or ligation arms, a logistic score >0.8, and successful amplification of loci. Marker loci and smMIP sequences are detailed in Table S2.

### 2.6 Oligonucleotide synthesis

smMIPs and primers for amplification and sequencing (Table S3), were synthesized by and purchased from Metabion.

### 2.7 smMIP phosphorylation and pooling

smMIPs were individually phosphorylated using 10 U of T4 Polynucleotide Kinase (NEB), 1X T4 DNA Ligase buffer (NEB), and 1 μM of unphosphorylated smMIP in a 100 µl reaction volume, and incubated at 37°C for 45 min, followed by 80°C for 20 min. Phosphorylated smMIPs were pooled, with specified volumes for each smMIP to equalize the number of reads from each marker locus, and diluted using Tris‐EDTA buffer (Sigma) to an average concentration of 0.1 nM (Table S4).

### 2.8 smMIP amplification

smMIP‐multiplexed amplification was based on Hiatt et al. (2013) using a SensoQuest thermocycler (SensoQuest GmbH), with minor modifications to the protocol. Herculase II Polymerase (Agilent) was used during extension and amplification steps for increased fidelity of microsatellite replication (Fazekas et al., 2010). For amplification, the thermocycler program used 98°C for 2 min, 30 cycles of 98°C for 15 s, 60°C for 30 s and 72°C for 30 s, followed by 72°C for 2 min. Hundred nanograms of sample DNA was used as a template unless stated otherwise: The input quantity of CRC sample DNA varied depending on quantity available (Table S1). smMIP reaction products (240–270 bp) were analyzed using 3% agarose gel electrophoresis at 80 mV for 60 min, or a QIAxcel (Qiagen).

### 2.9 Library preparation and sequencing

smMIP amplicons were purified using Agencourt AMPure XP Beads (Beckman Coulter), diluted to 4 nM in 10 mM Tris pH 8.5, and pooled in equal volumes. Libraries were sequenced on a MiSeq (Illumina) using the Generate FASTQ workflow, paired‐end sequencing, and custom sequencing primers (Hiatt et al., 2013); sequencing run statistics are presented in Table S5. FASTQ files are available from the EMBL‐EBI European Nucleotide Archive, accession number PRJEB28394.

### 2.10 Sequence analysis and MSI classification

Sequence analysis and MSI classification were carried out as described in by Redford et al. (2018). In brief, sequencing reads were aligned to the hg19 reference genome using the command BWA MEM (BWA v0.6.2; Li & Durbin, 2010). smMIP‐based sequencing assesses the regions of interest in both orientations, and only base calls supported by both reads of a pair were processed further. The MSI classifier uses both the frequency and allelic bias of deletions in the microsatellite markers to type each sample. The deletion frequency was defined as the proportion of reads that have a microsatellite length less than the reference length. For samples heterozygous at the neighboring SNP, the allelic bias of deletions, that is, whether deletions are preferentially observed in reads carrying one of the SNP alleles, can be assessed using the Fisher's Exact test *p* value. For each marker, deletion frequency and allelic bias were dichotomised into two binary traits; deletion frequency is assessed by whether it is above or below the 95th percentile of the training MSS samples, and allelic bias is assessed by whether the *p* value is above or below .05. A training cohort of samples was used to estimate the probabilities of observing the different traits for each marker in MSI‐H and MSS tumors. The (posterior) probability that a new sample is MSI‐H versus MSS can then be estimated from its microsatellite deletion frequencies, and the allelic bias of deletions, using a naïve Bayes approach. A prior probability of .85 that a sample is MSS was used. The assay score represents the decadic logarithm of the odds a sample is MSI‐H versus MSS. Scores >0 classify a sample as MSI‐H, and scores <0 classify a sample as MSS.

### 2.11 Statistics and graphics

Analyses were performed in R v3.3.1, and graphs generated with R package ggplot2. Scripts are available on request.

## 3 RESULTS

### 3.1 MSI classification is accurate and reproducible

**Figure 1:**
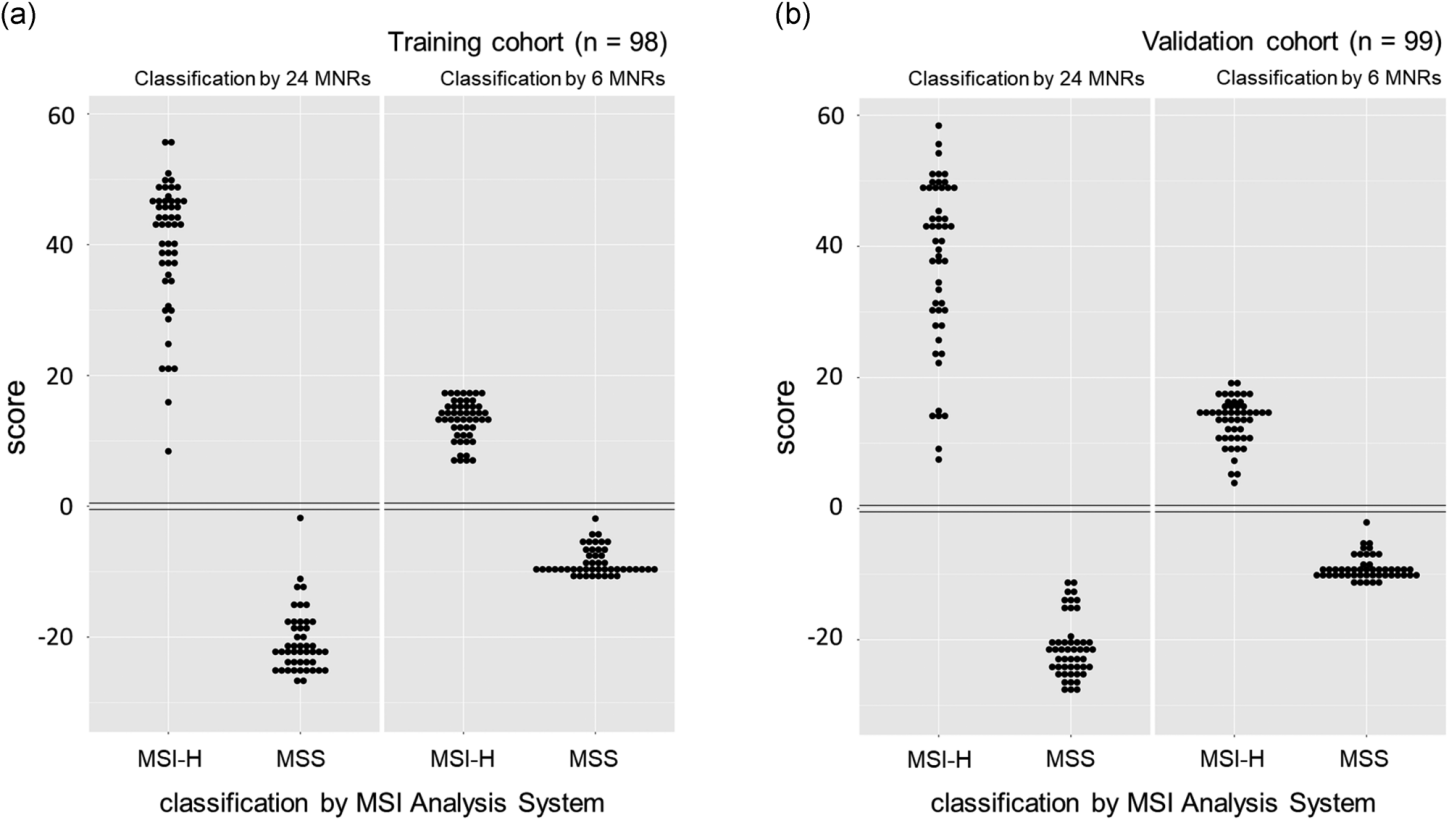
MSI classification of CRCs. MSI classifier scores versus diagnosis by the MSI Analysis System v1.2 (Promega) for CRCs analyzed in (a) the training cohort, and (b) the validation cohort. CRC, colorectal cancer; MNR, mononucleotide repeat; MSI, microsatellite instability; MSI‐H, high levels of MSI

smMIPs were designed for the 17 short MNR markers previously described in the singleplex assay of Redford et al. (2018). Two markers had to be excluded from the panel due to poor amplification by the smMIP protocol. To supplement this reduced panel, smMIPs were successfully designed for an additional nine markers taken from the extended set of Redford et al. (2018), giving a total of 24 short MNR markers (data not shown). Having defined the marker panel, a training cohort of 51 MSI‐H and 47 MSS CRCs was used to estimate classifier parameters. Reclassification of the training samples using these parameters achieved 100% sensitivity (95% confidence intervals [CIs]: 93.0–100.0%) and 100% specificity (95% CIs: 92.5–100.0%; Figure 1a). Data filtering using smMIP molecular barcodes to reduce sequencing error (Hiatt et al., 2013) did not improve sample separation by the classifier (Supporting Information S1), and therefore was not employed for MSI classification. The 15 markers remaining from the 17‐marker panel of Redford et al. (2018) also achieved 100% sensitivity and specificity (data not shown), indicating redundancy in the marker panel. Backward–forward stepwise selection was used to define a subset of six short MNRs (Table S2) with accuracy equal to the 24 marker panel (Figure 1A).

An independent validation cohort of 50 MSI‐H and 49 MSS was sequenced and analyzed, and 100% sensitivity (95% CIs: 92.9–100.0%) and 100% specificity (95% CIs: 92.8–100.0%) was again achieved using all 24 markers, and the 6‐marker subset (Figure 1b). To assess assay reproducibility, 16 MSI‐H and 16 MSS CRCs from the validation cohort were amplified, sequenced, and classified a second time. The classification was 100% concordant, and scores were strongly correlated between sample repeats, using both 24 markers (*β* = .97, *R* ^2^ = .97), and the 6‐marker subset (*β* = 1.01, *R* ^2^ = .97).

### 3.2 *BRAF* c.1799T>A variants are accurately detected

Independent testing of 46 MSI‐H CRCs for *BRAF* mutation by HRM had previously identified 14 samples positive for the c.1799T>A variant. By inclusion of a smMIP targeting *BRAF* in the assay, these 14 samples were found to have c.1799T>A variant allele frequencies (VAFs) >5%, a threshold generally considered to be clinically relevant in cancer diagnostics (Jennings et al., 2017). Of the 32 CRCs that tested negative, 30 had VAFs ≤0.60%, in line with observed MiSeq base‐calling error rates of 0.62% (May et al., 2015). The remaining two samples had VAFs of 1.67% and 1.72%, suggesting they may contain low‐frequency variants below the detection limit of HRM (Nikiforov et al., 2009). Consistent with this, when molecular barcodes were used to reduce sequencing error (Hiatt et al., 2013), these variants were found at frequencies of 1.82% and 0.46%, respectively (Table S6). This analysis identified an additional negative sample with a *BRAF* c.1799T>A VAF of 0.15%. Concordance (100%) was also observed for *BRAF* mutation calling in repeat testing of the 16 MSI‐H and 16 MSS CRCs, with a strong correlation of VAFs (*β* = .93, *R* ^2^ = 1.00).

### 3.3 MSI classification detects 3% MSI‐H cell line DNA in sample mixtures

**Figure 2:**
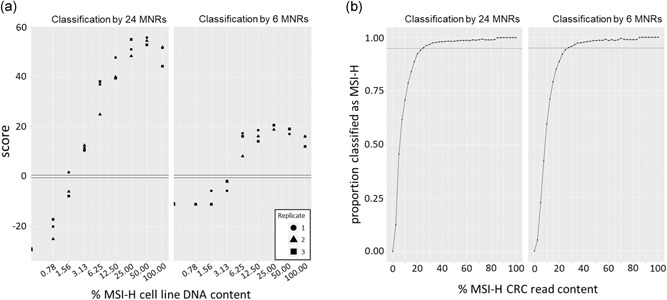
Assay robustness to sample heterogeneity. A, Classifier scores from mixtures of MSI‐H cell line and MSS PBL DNA samples. B, The proportion of correctly classified samples from 2400 simulated mixture series from the validation cohort reads (dotted line = 0.95). CRC, colorectal cancer; MNR, mononucleotide repeat; MSS, microsatellite stable; MSI‐H, high levels of MSI; PBL, peripheral blood leukocyte

To estimate the minimum MMR deficient tumor cell content required for a CRC to be classified as MSI‐H, we mixed DNA from HCT116 (a clonal, MMR deficient CRC cell line) with DNA from nonneoplastic PBLs to create, in triplicate, sample mixtures containing 0.78–100% DNA from MSI‐H cells. Across the mixtures, the observed and the theoretically expected proportion of reads containing insertion–deletion mutations in each microsatellite were strongly correlated (*β* = 1.03, *R* ^2^ = .99; Supporting Information S2), giving confidence in mixing accuracy. Mixtures containing ≥3.13% and ≥6.25% DNA from MSI‐H cells were classified as MSI‐H using the 24‐ and 6‐marker sets, respectively (Figure 2a), results which are better than or equivalent to FLA (Table S7).

We further investigated the impact of sample heterogeneity on classification in silico by randomly selecting sequencing reads from MSI‐H and MSS samples and mixing them in predetermined proportions to create simulated samples (Supporting Information S2). Scores from simulated samples were strongly correlated with scores from the mixing of DNAs (*β* = .97, *R* ^2^ = .98). Mixing reads from all pairwise combinations of MSI‐H and MSS samples from the validation cohort revealed that >95% of mixtures containing ≥25.0% reads from an MSI‐H CRC were classified as MSI‐H using the 24 marker panels, while ≥27.5% of MSI‐H CRC reads were needed to achieve the same level of classification accuracy using the 6‐marker panel (Figure 2b). As reads from MSI‐H CRCs are derived from heterogeneous mixtures of the tumor and normal tissue, this supports the conclusion that the MSI classifier is robust to low MMR deficient tumor cell content.

### 3.4 MSI classification is accurate when 10 or more molecules are sequenced per marker

**Figure 3:**
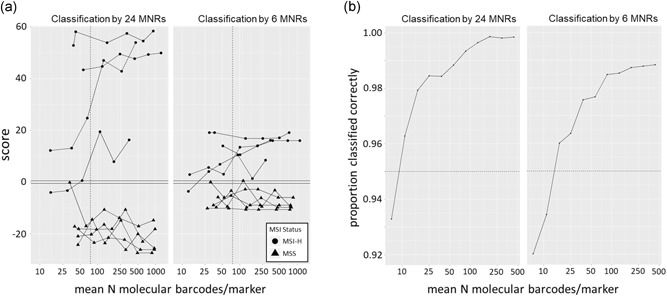
Assay robustness to variation in the quantity of sample DNA. A, Classifier scores from a serial dilution of nine samples, using 3.13–100 ng of template DNA (dotted line = 75 molecular barcodes per marker). B, The proportion of correctly classified samples from 60 simulated dilution series per sample in the validation cohort (dotted line = 0.95). MSS, microsatellite stable; MSI‐H, high levels of MSI; MNR, mononucleotide repeat

Whilst we found no improvement to classifier performance using molecular barcodes to correct sequencing error (Supporting Information S1), molecular barcodes can be used to estimate the number of template molecules sequenced to provide a quality control metric (Jennings et al., 2017). To establish this, and investigate the relationship between the number of template molecules sequenced and the accuracy of classification, we created twofold dilution series of nine samples, with template quantities ranging from 3.13 to 100 ng of DNA per reaction. A strong correlation between the input quantity of template DNA and the number of molecular barcodes detected across the nine samples (*R* ^2^ = .99–1.00; Supporting Information S3) confirmed the accuracy of dilution. Using the 24‐marker panel, all samples with a mean molecular barcodes per marker ≥75 were correctly classified, and among these there was no correlation between the number of molecular barcodes detected and any change in classifier score, relative to the baseline score from 100 ng of template DNA (*R* ^2^ = .10, *p* = .09; Figure 3a). However, below 75 molecular barcodes per marker, there was marked variability in the score for three samples (Figure 3a). Results were similar using the 6‐marker subset, except that one MSS sample with a mean molecular barcode detected per marker ≥75 was misclassified (Figure 3a). In agreement with these estimates, only one sample from the training and validation cohorts had a mean molecular barcode detected per marker <75 (Table S1), and all were correctly classified with either the 24‐ or 6‐marker panels.

To explore the minimum number of template molecules that need to be sequenced for accurate classification, we also performed an in silico resampling of sequencing data. Analysis of the nine‐sample dilution series gave a strong correlation between classifier scores from empirical observations and from resampling (*β* = .92, *R* ^2^ = .96; Supporting Information S3). Resampling of the CRCs included in the validation cohort was used to increase the number of observations, and it was found that using the 24‐ and 6‐marker panels, sequencing of ≥10 and ≥15 molecular barcodes per marker, respectively, gave a correct classification of >95% of samples (Figure 3b). It should be noted that these estimates were obtained from resampling high‐quality sequencing data. Therefore, the mean of 75 molecular barcodes per marker obtained empirically provides a more conservative threshold for diagnostic use.

### 3.5 Validation in an independent clinical laboratory

**Figure 4:**
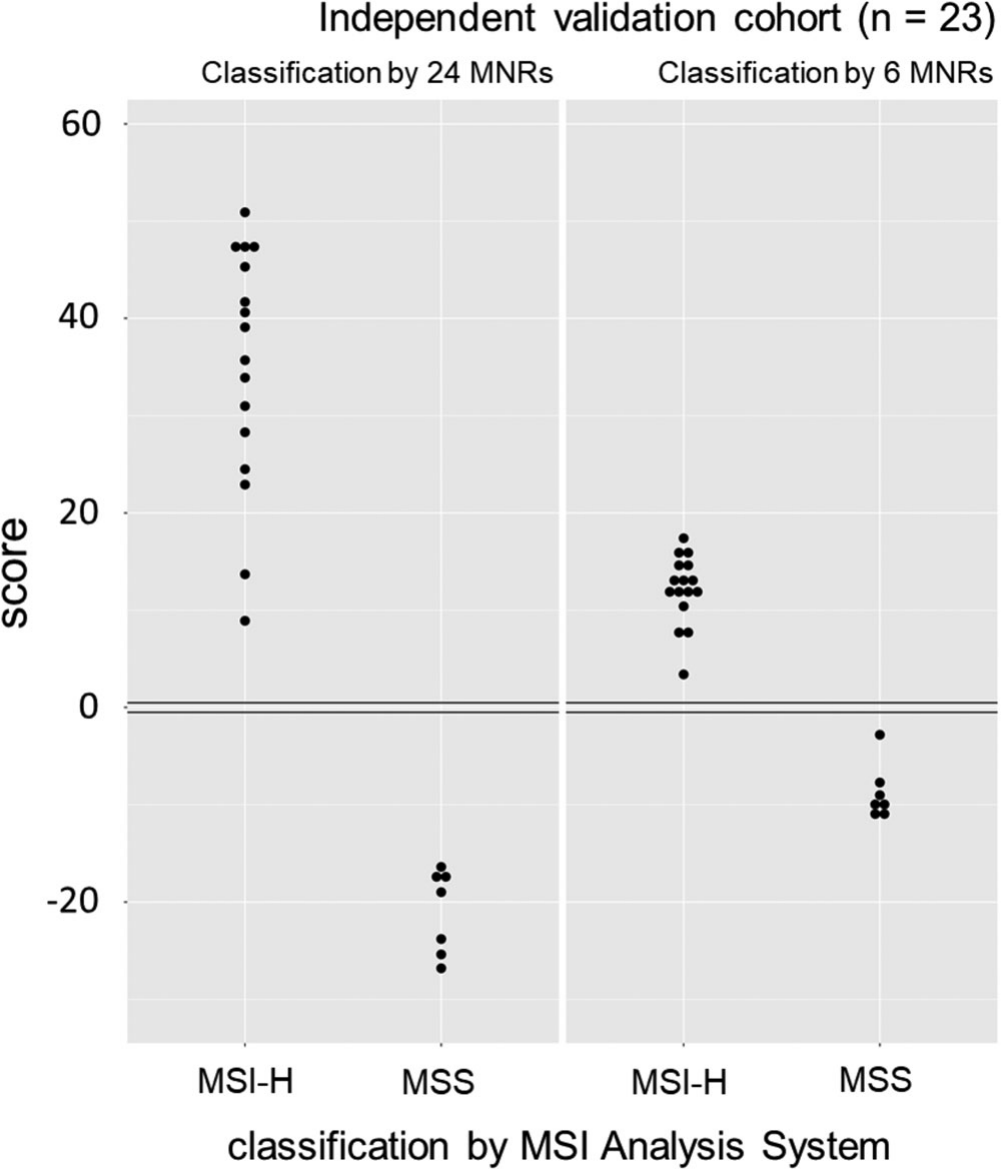
Assay validation in an independent laboratory. MSI classifier scores versus diagnosis by the MSI Analysis System v1.2 (Promega) for 23 CRCs tested by the Northern Genetics Service (Newcastle Hospitals NHS Foundation Trust, Newcastle, UK). CRC, colorectal cancer; MSI, microsatellite instability; MSI‐H, high levels of MSI

Assessment of an assay's performance in an independent clinical laboratory supports that it is a reproducible method, suitable for wider adoption (Jennings et al., 2017). To test this, our smMIP‐based MSI assay was set up by the Northern Genetics Service (Newcastle Hospitals NHS Foundation Trust, Newcastle upon Tyne, UK) using our protocols, and our smMIP and primer oligonucleotides. All other reagents and equipment were distinct from those used during assay development, and the personnel running the assay were independent of our research team. Once established, a further 23 independent CRCs were analyzed using the assay, and it again achieved 100% sensitivity (95% CIs: 79.4–100.0%) and 100% specificity (95% CIs: 59.0–100.0%) relative to the MSI Analysis System v1.2, when classifying samples with both the 24‐ and 6‐marker panels (Figure 4). Although four samples had <75 molecular barcodes per marker detected (Table S1), they were accurately classified in agreement with reading sampling predictions, and so were not resequenced at a higher depth.

## 4 DISCUSSION

The MSI assay presented here achieved 100% accuracy of MSI classification in 220 CRCs, relative to the MSI Analysis System v1.2 (Promega), using only tumor DNA and as few as six microsatellite markers. We found no improvement to classifier performance using molecular barcodes for sequencing error correction (Hiatt et al., 2013; Supporting Information S1). This is likely due to our use of short MNRs with flanking SNPs, selected from genome‐wide data, and classification method. Shorter microsatellites have lower PCR and sequencing error rates compared with longer microsatellites (Fazekas et al., 2010), while the SNPs flanking the microsatellites provide additional discrimination between error and true microsatellite mutations. Classification by a naïve Bayesian approach accounts for individual marker sensitivity, specificity, and sequencing error rate (Redford et al., 2018). However, molecular barcodes are used in our assay to provide a quality control metric by estimating the number of independent molecules sequenced (Jennings et al., 2017). We have also shown, previously, that molecular barcodes are useful for the detection of much lower frequency microsatellite variants, found in the PBLs of patients with constitutional mismatch repair deficiency (CMMRD; Gallon et al., 2019).

To show that the assay is suitable for clinical practice, we tested its clinical validity according to published guidelines (Jennings et al., 2017). We validated its accuracy (100%) across three cohorts of clinical samples, which included poor‐quality DNA samples from FFPE tissue, and 23 CRCs analyzed by an independent diagnostic laboratory. We also observed 100% classification concordance in repeat testing and assessed robustness to sample heterogeneity using sample mixing, detecting 3% and 6% MSI‐H cell line DNA with the 24‐ and 6‐marker panels, respectively. Depending on the marker panel used, we estimate that 10 or 15 molecular barcodes per marker are required for correct classification of >95% of samples. We have, therefore, shown that it is possible to accurately determine MSI status using only six markers, a fraction of the number required by other NGS‐based MSI assays (Kautto et al., 2016; Waalkes et al., 2018; Zhu et al., 2018), and observed only a small reduction in assay robustness using this subset rather than the 24‐marker panel. The requirement of other NGS‐based MSI classifiers for larger marker panels may be explained by the classification method, as assessing the proportion of mutated microsatellites gives equal diagnostic weight to each marker and does not account for the variable influence of MMR deficiency on the mutation of individual microsatellites (Dietmaier et al., 1997).

Marker number has a significant impact on cost, and with only six markers, plus *BRAF* c.1799, our reagent cost estimates range from £5.55–6.81 per sample, depending on the capacity of the MiSeq kit used (Supporting Information S4). The 24 marker set may, however, be preferred for a variety of reasons: It could provide protection against allele or marker drop out due to technical variation, somatic events within tumors, or population‐specific sequence variants. It may also enhance the clinical utility of the assay, as it increases the power of the internal sample traceability provided by the SNPs linked to each marker. For instance, using the allele frequencies observed in the training cohort, the probability of any two individuals sharing the same genotype is 3.8 × 10^−3^ from the 6‐marker subset, but 3.6 × 10^−10^ when 24 markers are used (Table S2).

The clinical demand for MSI analysis may increase, driven by the need to predict patient response to immune checkpoint blockade therapy across multiple cancer types (Le et al., 2017). The frequency of mutations in noncoding microsatellites has been shown to be equivalent between different cancer types (Cortes‐Ciriano, Lee, Park, Kim, & Park, 2017), and we have previously shown that the 24 markers analyzed here can detect CMMRD from PBL DNA (Gallon et al., 2019), making it likely that our assay will be suitable for MSI detection in extra‐colonic tissues. For the identification of LS through screening all CRCs for MMR deficiency, the inclusion of *BRAF* c.1799 avoids expenditure on additional tests as a single tumor assay is required before germline testing. It also demonstrates the modularity of the assay, which can be expanded to cover additional clinically relevant markers, or adapted to different tumor types, with ease since thousands of smMIPs can be multiplexed (Hiatt et al., 2013; Oud et al., 2017). *MLH1* promoter methylation is an alternative marker to the *BRAF* c.1799T>A variant to exclude sporadic MMR deficient patients with CRC from germline testing and has superior specificity for LS detection (Pérez‐Carbonell et al., 2010). However, this also excludes *MLH1* mutation carriers who have methylation as the second hit in their tumor (Moreira et al., 2015), or have germline epimutations (Suter, Martin, & Ward, 2004).

In summary, the MSI assay outlined here is accurate, reproducible, robust to sample heterogeneity, and includes both internal quality controls and sample identification. The automatable laboratory workflow and analysis, and the need for as few as six microsatellite markers at moderate read depths provide a cheap and scalable option for high‐throughput MMR deficiency testing.

## CONFLICT OF INTERESTS

LR, GA, MSK, MSJ, and JB are named as inventors on the patent held by their employer Newcastle University covering the markers used in this assay (Patent ID: PCT/GB2017/052488, published March 1, 2018). In recognition of early support and release of intellectual property, QuantuMDx Ltd will receive a percentage of any income from commercialization. RG, HS, MSK, MSJ, and JB are named as inventors, and CH is named as a contributor, on an additional patent filed by their employer Newcastle University covering the reduced marker set described in this paper (PCT application number: PCT/GB2019/052148, unpublished, filing date July 31, 2019). JB receives an annual salary from QuantuMDx Ltd as their Chairman. JB and family members are shareholders in QuantuMDx Ltd. All other authors have no conflict of interests to declare.

## Supporting information

Supporting information

Supporting information

Supporting information

Supporting information

Supporting information

Supporting information

Supporting information

Supporting information

Supporting information
