# Supplementary material for: Sequencing‐based microsatellite instability testing using as few as six markers for high‐throughput clinical diagnostics
Source: Hum Mutat. 2019 Sep 15;41(1):332–41. doi: 10.1002/humu.23906 (PMC6973255; doi:10.1002/humu.23906)
Supplement: Supplementary file 9 — Supporting information [file HUMU-41-332-s009.pdf]

Supp. Information S1: Sequencing error reduction by use of molecular barcodes does not improve sample classification

Molecular barcodes can be used to reduce PCR and sequencing error. In brief, reads that share the same molecular barcode are assumed to originate from one template DNA molecule, and, within each molecular barcode group, any PCR and sequencing errors are assumed to occur in the minority of reads. Based on these assumptions, a single molecule sequence (smSequence), which represents the true sequence of the position or feature of interest in the template DNA molecule, can be defined from the sequence represented in the majority of reads of each molecular barcode group containing a minimum of 2 reads. Groups with single reads are discarded. We have shown that smSequences have much lower error rates for microsatellite variant detection than sequencing reads not processed by molecular barcodes. smSequences of microsatellites can, therefore, be used to detect low frequency microsatellite variants in normal tissues to diagnose constitutional mismatch repair deficiency, a rare childhood cancer syndrome (Gallon *et al*, 2019; doi: 10.1002/humu.23721).

We assessed if the use of molecular barcodes could improve the performance of the MSI classifier for CRC diagnostics. As described above, the method requires redundant sequencing of the template DNA molecules. Within the training cohort (n = 98), a mean 1139 reads were sequenced per marker per sample, with a mean 380 molecular barcodes detected per marker per sample (Supplementary Table S1). Therefore, on average, each molecular barcode group contained 3.00 reads, allowing smSequences to be used to train the classifier by the method of Redford *et al* (2018; doi:10.1371/journal.pone.0203052). We used three measures to analyse classifier performance: diagnostic accuracy, difference

between the median scores of MSI-H versus MSS CRCs, and the difference between the minimum MSI-H sample score and maximum MSS sample score. The smSequence-trained classifier classified the training cohort with 100% accuracy (Figure S1.1A), equal to the read-trained classifier. However, it gave slightly poorer separation of MSI-H from MSS samples when compared to the read-trained classifier, with a reduction in the difference between median scores of MSI-H and MSS samples, and a reduction in the difference between the minimum MSI-H score versus maximum MSS score (Table S1.1). The validation cohort (n = 99) had a mean 8.40 reads per molecular barcode, and could, therefore, be classified by the smSequence-trained classifier. Again, the smSequence-trained classifier had 100% diagnostic accuracy (Figure S1.1B), as for the read-trained classifier, but gave poorer separation of MSI-H from MSS CRCs (Table S1.1).

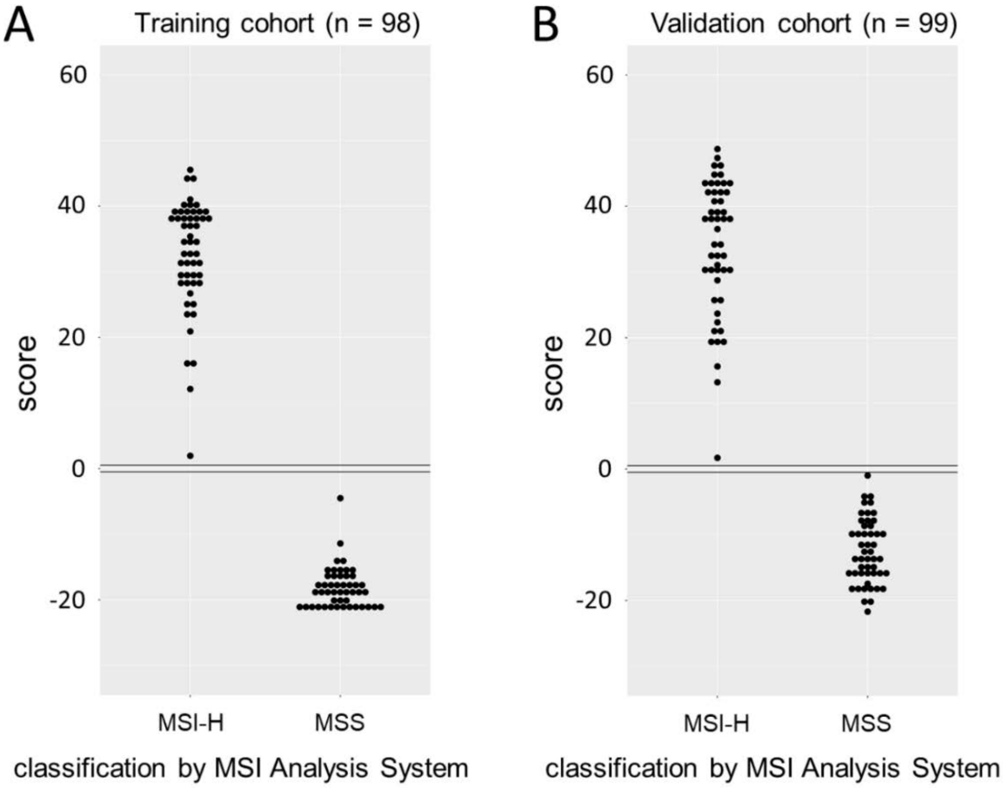

Figure S1.1: Classification of the training **(A)** and validation **(B)** cohorts by the smSequence-trained MSI classifier.

Cohort	Score comparator	Classifier method	Score difference
Training	Median	By Reads	65.3
Training	Median	By smSequences	53
Training	Minimum MSI-H vs. maximum MSS	By Reads	10.2
Training	Minimum MSI-H vs. maximum MSS	By smSequences	6.5
Validation	Median	By Reads	62.7
Validation	Median	By smSequences	50.3
Validation	Minimum MSI-H vs. maximum MSS	By Reads	18.5
Validation	Minimum MSI-H vs. maximum MSS	By smSequences	2.7

Table S1.1: Comparison of the difference in classifier scores of MSI-H and MSS CRC samples by the read- or smSequence-trained MSI classifier, in the training and validation cohorts.

In conclusion, it was decided that molecular barcodes should not be used to reduce sequencing error prior to sample classification due to the equal diagnostic accuracy, but worse sample separation, achieved by an smSequence-trained classifier versus a read-trained classifier. That we did not observe improved classification by use of molecular barcodes is likely due to the naïve Bayesian approach used to train the classifier. In particular, the effect of sequencing error is incorporated into the probabilities used within the classifier parameters, and this will reduce the impact of error reduction by use of molecular barcodes. The worse separation of MSI-H from MSS samples could result from the reduced quantity of data per marker per sample used for classifier training because the generation of smSequences discards data, such as molecular barcode groups containing only 1 read. It is possible that an smSequence-trained classifier could have equivalent, or superior, separation of MSI-H from MSS samples if higher read depths and more samples were used. However, optimising classifier training was not deemed necessary for this study given its 100% diagnostic accuracy using the read-trained classifier, and a requirement for higher read depths would obstruct our aim of a cheap and scalable assay.

Supp. Information S2: DNA and read mixing series

Assessing the effect of DNA mixing

Assuming there is no selective amplification of microsatellite alleles from MSI-H or MSS cells, a comparison of the observed relative frequency of microsatellite length mutations in DNA mixtures of HCT116 (MSI-H cell line) and PBLs (MSS cells) can be compared to the expected frequency (f^{mix}) to assess the accuracy of mixing. f^{mix} was calculated from the mean frequencies of microsatellite length mutations observed from HCT116 DNA (f^{MSI}) and PBLs (f^{MSS}), and the proportion of MSI-H cell DNA in the mixture (p^{mix}), using the equation:

$$f^{mix} = p^{mix}.f^{MSI} + (1 - p^{mix}).f^{MSS}$$

The observed frequencies were strongly correlated with expected frequencies (β = 1.03, R^2 = 0.99, Figure S2.1).

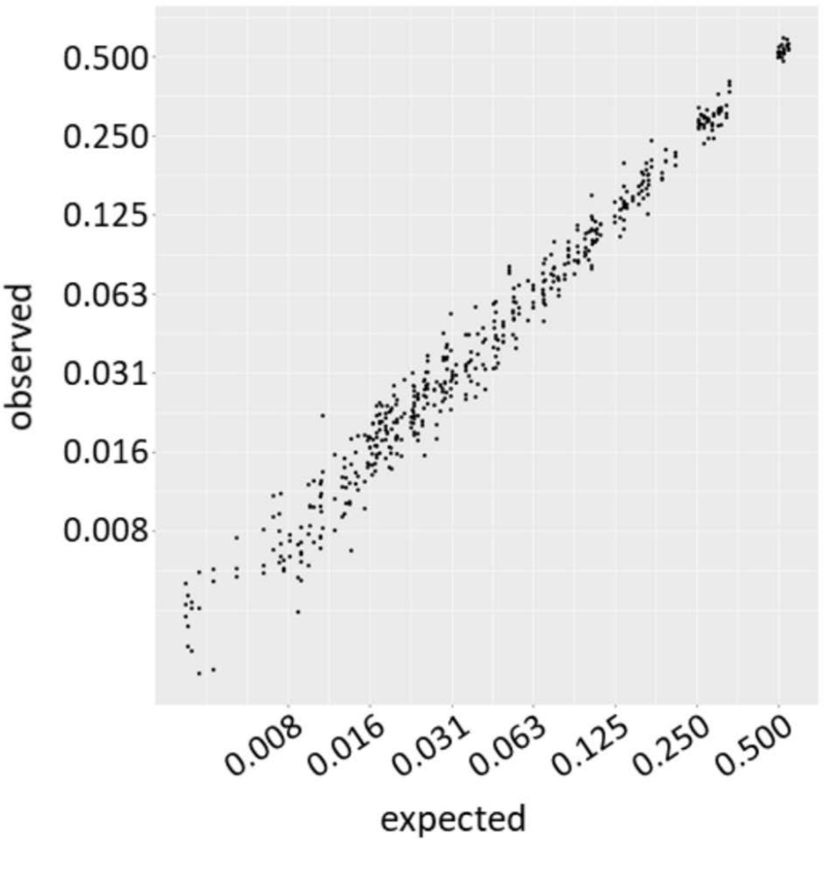

Figure S2.1: Observed versus expected relative frequency of microsatellite length mutations in the three replicate sample mixture series, ranging from 0.78-50.00% MSI-H cell DNA.

Simulating additional samples of varying MSI-H cell content

Additional samples of varying MSI-H content were simulated by mixing reads from one MSI-H sample and one MSS sample. For each marker in a simulated sample, reads were randomly mixed in the desired proportion, with the total number of reads per marker equal to the reads per marker of the MSI-H sample used. Classifier scores of simulated and empirical sample mixtures were strongly correlated using data from HCT116 and PBLs (β = 0.97, R^2 = 0.98; Figure S2.2), supporting the validity of the method.

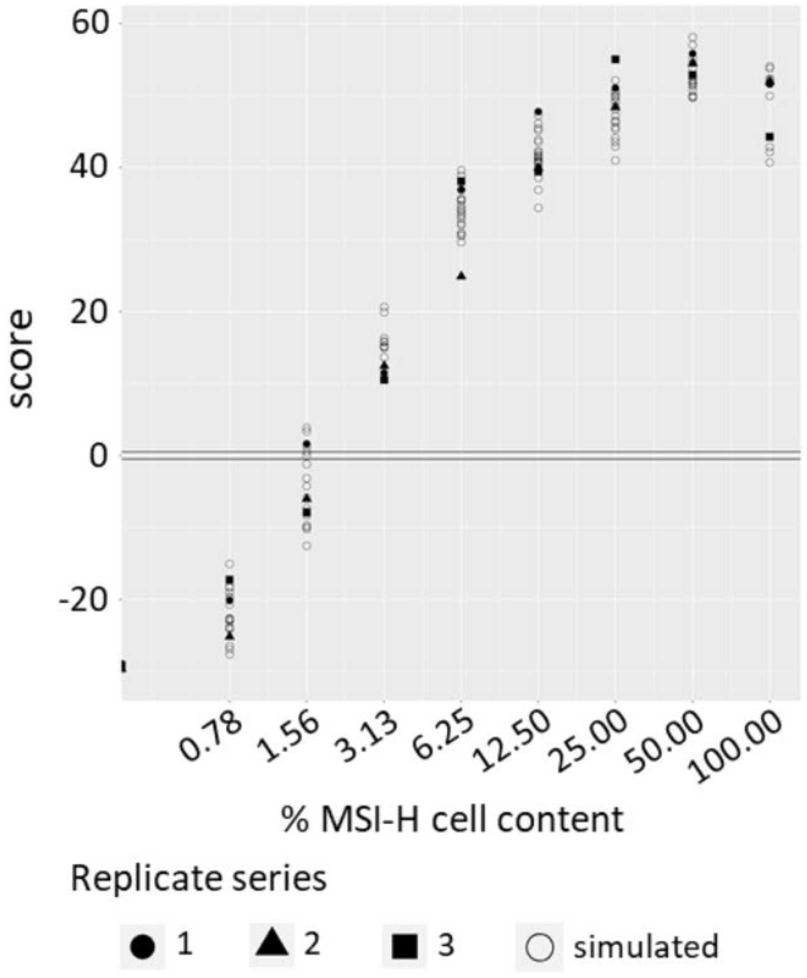

Figure S2.2: Classifier scores of simulated and empirical sample mixture series, ranging from 0.00-100.00% MSI-H cell DNA.

Supp. Information S3: DNA and read dilution series

Visualisation of amplicons from the template DNA dilution series (Figure S3.1)

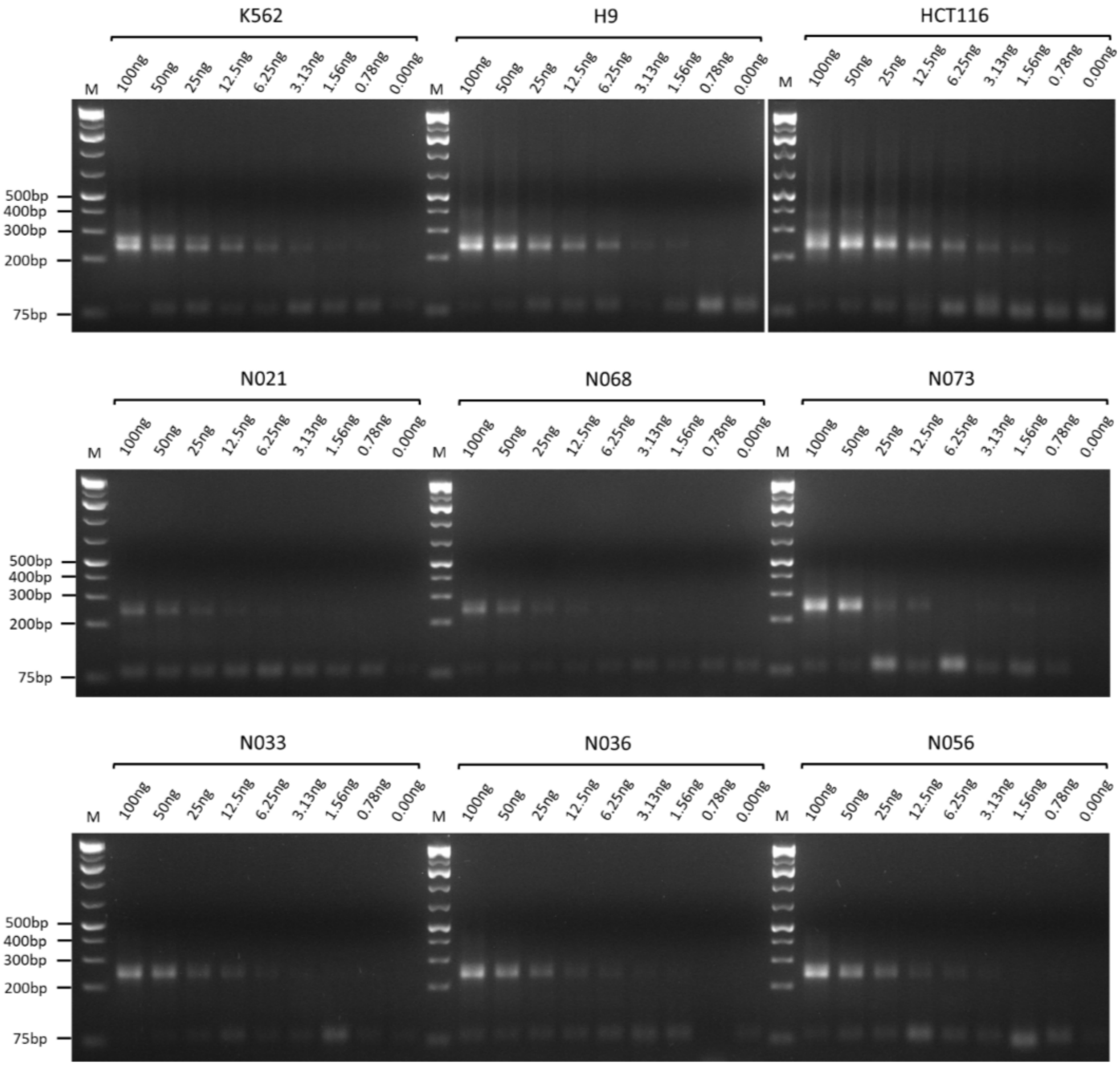

Figure S3.1: Agarose gel electrophoresis of amplicons generated from 9 samples by the MSI assay. Amplicons are visible at 240-270bp. Primer dimers are visible at 80bp. Marker (M): GeneRuler 1kb Plus (ThermoFisher). Top panel: cell line controls. Middle panel: FFPE MSI-high CRCs. Bottom panel: FFPE MSS CRCs.

Assessing the effect of sample dilutions on the number of molecular barcodes detected

Assuming that each molecular barcode originates from a distinct template DNA molecule, the number of molecular barcodes detected can be used to assess the accuracy of the sample dilution series. The number of molecular barcodes detected was correlated with the quantity of template DNA for each of the 9 samples (β = 0.84-0.96, R^2 = 0.99-1.00, Figure S3.2), suggesting dilutions were accurate. One sample, N073, shows a lower than expected number of molecular barcodes from 6.25ng of template DNA, which is also visible from a reduced intensity of amplicon in the gel image (Figure S3.1), suggesting there was an error in reaction preparation.

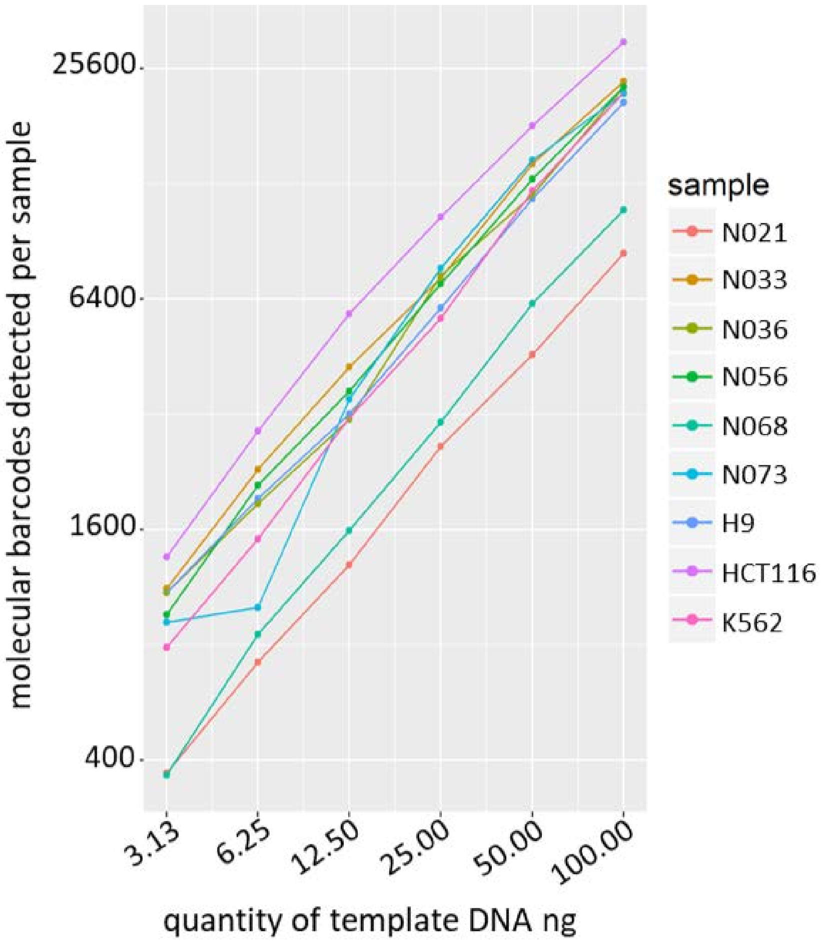

Figure S3.2: Correlation of the number of molecular barcodes detected, and the input quantity of template DNA.

Simulating sample dilutions to decrease molecular barcodes detected

Additional sample dilution series were simulated by resampling of reads. For each marker in a sample, reads were grouped by molecular barcode, and the microsatellite length and SNP genotype associated with that molecular barcode was summarised from that found in the majority of reads in the group. Predetermined numbers of molecular barcodes were selected to simulate sample dilution. Subsequently, reads for the simulated sample were generated to a depth equal to that of the original sample, with each read having a defined microsatellite length and SNP genotype by random sampling of the selected molecular barcodes.

For each of the 9 samples that were empirically analysed, 20 simulated dilution series were generated, using 7, 11, 17, 25, 38, 57, 86, 129, 194, 291, 437, 656 and 985 molecular barcodes per marker, and classifier scores were modelled by non-linear regression using cubic splines, such that a simulated score could be predicted for any given number of molecular barcodes per marker. Classifier scores of simulated and empirical sample dilutions, of the same mean number of molecular barcodes per marker, were strongly correlated using data from the 9 samples ($\beta = 0.92$, $R^2 = 0.96$; Figure S3.3), supporting the validity of the method.

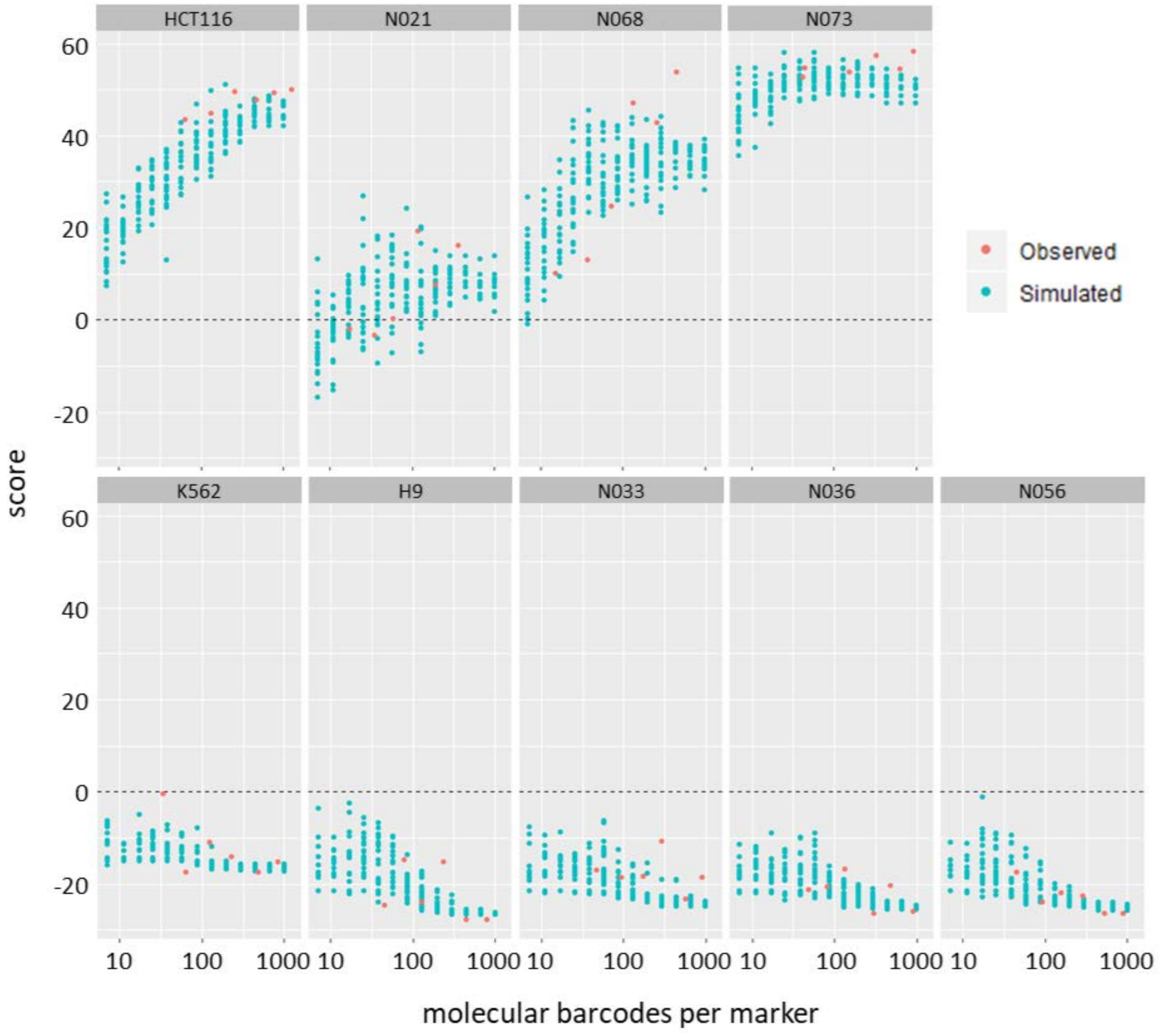

Figure S3.3: Comparison of empirically observed and simulated sample dilution series, and the association between molecular barcodes/marker and classifier score.
